# Nanoclay- and alginate-based soil amendments preserve photosynthetic function, delay senescence, and reduce oxidative damage in drought-stressed wheat

**DOI:** 10.3389/fpls.2026.1842102

**Published:** 2026-07-27

**Authors:** Ali El-Keblawy, Abdelaziz Elgamouz, Mohamed Abdallah, Mohamed G. Arab, Khalifa M. Alteneiji, Razan F. Alhammadi, Lamya Alsuwaidi, Walid Abo Baker, Mohammad A. Al-Faqieh, Soumya Koippully Manikandan

**Affiliations:** 1 Department of Applied Biology, University of Sharjah, Sharjah, United Arab Emirates; 2 Advanced Biotechnology Center, Research Institute of Sciences and Engineering, University of Sharjah, Sharjah, United Arab Emirates; 3 Faculty of Pharmacy, Alsalam University, Tanta, Egypt; 4 Department of Chemistry, College of Science, University of Sharjah, Sharjah, United Arab Emirates; 5 Department of Civil and Environmental Engineering, University of Sharjah, Sharjah, United Arab Emirates; 6 Agriculture and Livestock Department, Sharjah Government, Sharjah, United Arab Emirates

**Keywords:** antioxidant enzymes, photosystem II, proline, reactive oxygen species, redox metabolism, water deficit

## Abstract

**Introduction:**

Drought stress accelerates leaf senescence, disrupts photosynthetic function, and enhances oxidative damage in wheat, yet the extent to which soil amendments can mitigate these responses remains insufficiently understood. This study examined whether nanoclay (CN), calcium alginate (CG), and nanoclay–calcium alginate composite formulations (CNG) could modulate senescence, redox status, and photosynthetic performance under drought conditions.

**Methods:**

A greenhouse experiment was conducted using wheat grown under regular watering and drought stress conditions. Plants were treated with CN, CG, and CNG composite formulations, and responses were assessed through physiological and biochemical traits related to senescence, chlorophyll status, photosystem II (PSII) performance, oxidative stress, antioxidant enzyme activity, and proline accumulation. Principal component analysis (PCA) was performed to evaluate multivariate treatment effects.

**Results:**

Drought caused pronounced physiological and biochemical deterioration in the untreated control, including increased leaf yellowing, chlorophyll depletion, reduced PSII performance, and elevated hydrogen peroxide (H_2_O_2_) and malondialdehyde (MDA) levels. Amendment effects were strongly trait dependent. CNG 31, particularly at 1.5%, most consistently delayed drought-induced senescence and reduced oxidative injury, whereas CG treatments were more effective in preserving chlorophyll content and photochemical performance. APX and GPX activities varied among formulations and did not show a uniform drought response, suggesting that mitigation involved differential regulation of redox metabolism rather than a simple increase in antioxidant activity. Proline accumulation was influenced mainly by water regime and showed weaker discrimination among treatments. Principal component analysis revealed clear multivariate separation among droughted treatment groups, reflecting coordinated variation in senescence, oxidative, and antioxidant traits.

**Conclusion:**

Nanoclay- and alginate-based amendments modulated wheat drought responses through coordinated effects on senescence progression, photosynthetic integrity, and oxidative balance, with formulation-specific differences in the dominant protective mechanisms. These findings highlight the potential of tailored soil amendments to improve wheat resilience under water-limited conditions.

## Graphical Abstract

**Figure:**
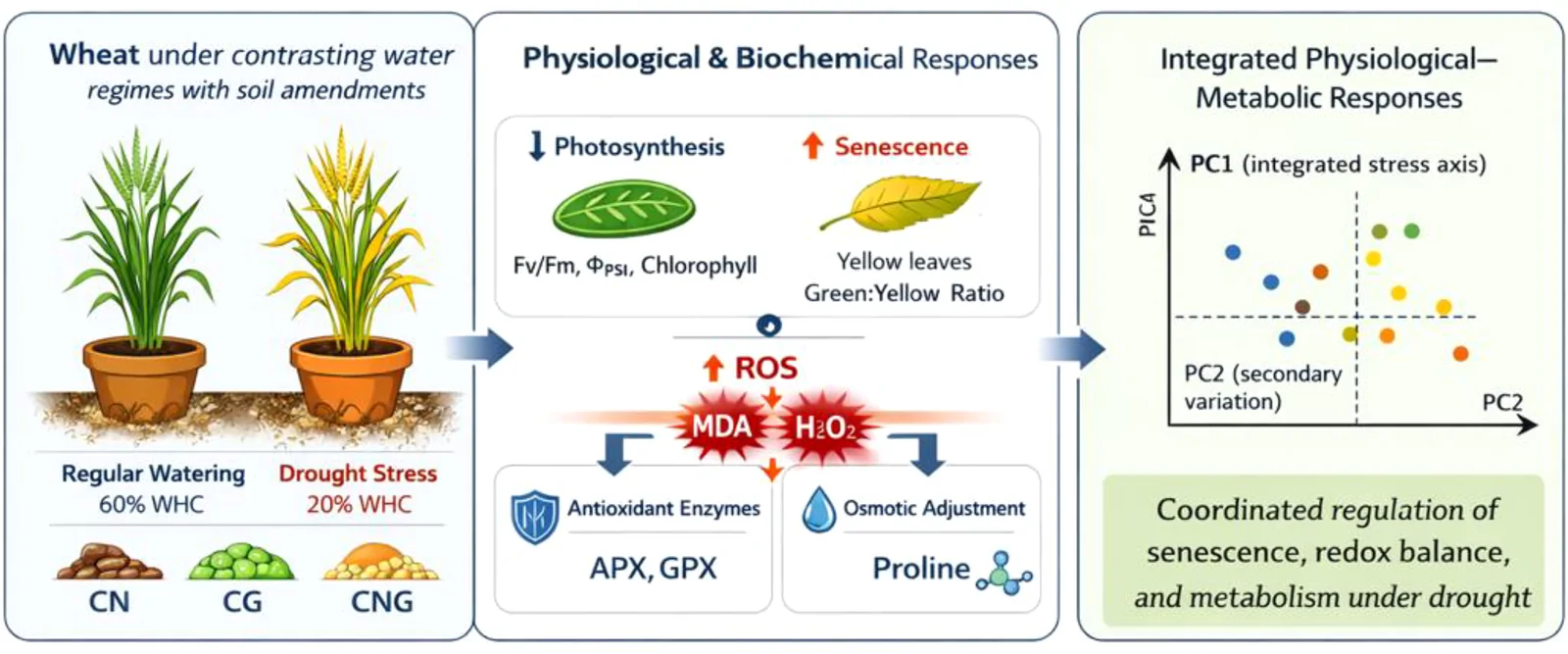

## 1 Introduction

Drought stress is a major constraint on wheat production, adversely affecting productivity through coordinated changes in leaf senescence, photosynthetic performance, oxidative balance, and metabolic adjustment. In cereals, water deficit commonly accelerates chlorophyll loss, impairs photosystem II (PSII) function, increases reactive oxygen species (ROS) accumulation, and alters antioxidant metabolism, ultimately shortening the duration of effective canopy function (Anderegg et al., 2021; Guo et al., 2021). The interconnected nature of these processes indicates that drought tolerance cannot be adequately explained by growth responses alone but requires integrated evaluation of senescence progression, photochemical performance, and redox regulation. Drought-induced metabolic imbalance increases ROS production, particularly in chloroplasts and peroxisomes, where ROS function as signaling molecules at low concentrations but promote oxidative damage and accelerated senescence when excessively accumulated (Liu et al., 2026). This oxidative dimension of drought stress is commonly evaluated using biochemical markers such as hydrogen peroxide (H_2_O_2_) and malondialdehyde (MDA), the latter being a widely used indicator of lipid peroxidation and membrane damage (Khan et al., 2026). Plants mitigate ROS accumulation through antioxidant and osmotic adjustment pathways; among these, ascorbate peroxidase (APX) is a major component of the ascorbate–glutathione cycle, guaiacol peroxidase (GPX) contributes to H_2_O_2_ detoxification and oxidative metabolism, and proline accumulation supports osmotic balance and stress adjustment, although the magnitude and coordination of these responses vary among genotypes and stress scenarios (Semida et al., 2020; Li, 2023; Talaat and Abdel-Salam, 2024).

Chloroplasts are both a major source and a primary target of drought-induced oxidative stress; therefore, assessment of photosynthetic integrity provides an important functional complement to biochemical stress markers (Razi and Muneer, 2021; Wang et al., 2025). Chlorophyll fluorescence measurements are widely used as non-destructive indicators of PSII performance, with F_v_/F_m_ reflecting the maximum photochemical efficiency of PSII in dark-adapted leaves and ΦPSII reflecting the effective photochemical efficiency of PSII under light (Lysenko et al., 2022; Li et al., 2026). In parallel, chlorophyll content provides a structural–functional readout of pigment stability that is often linked to drought-induced senescence. Leaf senescence is a regulated process accelerated by drought through chlorophyll degradation, chloroplast dismantling, and loss of functional green leaf area (Guo et al., 2021). Accordingly, senescence traits such as yellow leaf counts, fraction of yellow leaves, and green:yellow ratios can serve as integrative phenotypic indicators of drought stress when interpreted alongside photochemical and oxidative markers.

Soil amendments have increasingly been investigated as drought-mitigation tools because they can alter the soil–plant water environment and thereby modify the intensity and duration of stress experienced by plant tissues. Alginate-based hydrogels and composite soil conditioners have attracted attention because of their water-absorbing capacity, biodegradability, and potential to improve soil water retention and nutrient dynamics (Gomaa and Aldaby, 2023). Recent studies also demonstrate the feasibility of alginate materials in soil amendment systems, including alginate beads, slow-release constructs, and other composite conditioners relevant to drought-prone production environments (Anee et al., 2022; van der Merwe et al., 2022; Wang et al., 2023). In parallel, nanoclay-containing materials have attracted interest because layered silicates are abundant and can be incorporated into composites with tunable physicochemical properties. Although nanoclays may support water retention and soil conditioning when formulated appropriately, there is also evidence that clay nanoparticles can influence plant water relations through physical effects on root water transport, highlighting the need for plant-level physiological and biochemical evaluation rather than assuming consistent benefit (Asli and Neumann, 2009; Abd-Elsalam et al., 2024). This duality provides a strong rationale for mechanistic studies linking nanoclay-based amendments to oxidative stress, senescence progression, and photosynthetic integrity under drought conditions (Padil et al., 2022).

Despite the expanding materials literature, important knowledge gaps remain at the plant-stress physiology level. In wheat, comparatively few studies have integrated senescence phenotypes, chlorophyll fluorescence and pigment stability, and oxidative stress markers with antioxidant enzyme regulation and osmotic adjustment under controlled drought in systems treated with biopolymer–mineral amendment combinations. Consequently, the physiological mechanisms through which nanoclay–alginate amendments influence drought responses remain insufficiently understood.

Accordingly, the objective of this study was to evaluate how nanoclay (CN), calcium alginate (CG), and nanoclay–alginate composite formulations (CNG) influence wheat (Triticum aestivum L.) responses to drought stress at biochemical, physiological, and phenotypic levels. The evaluated parameters comprised senescence traits, including yellow leaf number, fraction of yellow leaves, and green:yellow ratio; photosynthetic parameters, including the maximum quantum efficiency of photosystem II (F_v_/F_m_), the effective quantum yield of photosystem II (ΦPSII), and total chlorophyll; oxidative stress indicators, including malondialdehyde (MDA) and hydrogen peroxide (H_2_O_2_); antioxidant enzyme activities, including ascorbate peroxidase (APX) and guaiacol peroxidase (GPX); and proline accumulation. The study tested the hypothesis that nanoclay–alginate composite formulations provide greater drought protection than the individual amendments by promoting coordinated regulation of oxidative stress, photosynthetic performance, and senescence under water-limited condition.

## 2 Materials and methods

### 2.1 Plant material and experimental design

A pot experiment was conducted using bread wheat (*Triticum aestivum* L., cv. Yecora Rojo) grown under controlled greenhouse conditions. Seeds were manually cleaned to remove surface coatings and hydro-primed in aerated distilled water for 48 h at room temperature to ensure uniform germination (Faisal et al., 2023). Seeds were sown in plastic pots containing 230 g of soil per pot and grown for a total of 60 days.

The experiment followed a factorial design with two fixed factors: water regime and treatment group, with five replicate pots per treatment–water-regime combination. Seven seedlings were initially established per pot and thinned to five uniform plants after emergence to minimize within-pot variability.

### 2.2 Soil amendments and treatment application

CN was prepared from locally sourced clay using date palm pit extract as a stabilizing and dispersing agent. The clay suspension was ultrasonicated to improve particle dispersion (Gdeh et al., 2026), then washed and dried. CG was prepared by ionic gelation of sodium alginate with calcium chloride (Li et al., 2016), and composite formulations (CNG 31, CNG 11, and CNG 13) were produced by combining CN and CG at the required CN: CG mass ratios using the same gelation approach. Selected physicochemical properties of CN, CG, and the composite formulations are summarized in **Supplementary Table 1**, including Brunauer–Emmett–Teller (BET) surface area, total pore volume, mean pore radius, and particle size. Amendments were applied at predefined rates (0.25–1.5% w/w, depending on the treatment) by thoroughly mixing them into the soil prior to sowing. Untreated soil served as control.

### 2.3 Watering regime and nutrient management

Soil water-holding capacity (WHC) was determined gravimetrically by measuring soil saturation and dry weight prior to the experiment (Verheijen et al., 2019). Two irrigation regimes were imposed: regular watering (R; 60% WHC) and drought stress (S; 20% WHC). All pots were maintained at 60% WHC during the initial establishment phase, after which drought stress was imposed on the stress treatments. Soil moisture was maintained throughout the experiment by gravimetric control, with pots weighed regularly and irrigated to target weights corresponding to the assigned water regime. At each weighing, the amount of water added to restore each pot to its assigned target weight was recorded. Cumulative recorded water addition was calculated as the sum of these top-up amounts over the experimental period and was used only for context regarding pot-scale water requirements. Evaporation, transpiration, and soil hydraulic properties were not partitioned or directly measured. To avoid nutrient limitation, all treatments received identical nutrient supplementation. Plants were supplied periodically with full-strength Hoagland nutrient solution (Hoagland and Arnon, 1950) and a water-soluble NPK fertilizer (20:20:20) was applied uniformly across treatments. This ensured that observed treatment effects were attributable to water regime and soil amendments rather than nutrient availability.

### 2.4 Senescence assessment

Leaf senescence was assessed at harvest based on visible chlorosis, a widely used indicator of drought-induced senescence in cereals (Anderegg et al., 2021). For each pot, leaves were visually classified as green (fully green lamina) or yellow (clear loss of green pigmentation), and the number of yellow leaves was recorded. The fraction of yellow leaves was calculated as the number of yellow leaves divided by the total number of leaves per pot. In addition, the green:yellow leaf ratio was calculated as a complementary index of senescence status. These metrics were used to quantify drought- and treatment-associated shifts in canopy senescence.

### 2.5 Chlorophyll fluorescence and pigment measurements

Chlorophyll fluorescence was assessed on the youngest fully expanded, healthy leaves using a pulse-amplitude modulated fluorometer (FMS-2, Hansatech Instruments Ltd., Norfolk, UK). Measurements were conducted during mid-morning to minimize diurnal variability (Lutz et al., 2025). Leaves were dark-adapted for 20 min using leaf clips prior to determination of the maximum quantum efficiency of PSII (F_v_/F_m_), calculated as (F_m_ − F_0_)/F_m_, where F_0_ is minimum fluorescence and F_m_ is maximum fluorescence measured after a saturating pulse. The effective quantum yield of PSII (ΦPSII) was determined under actinic illumination using the saturating pulse method as ΦPSII = (F_m_′ − F_s_)/F_m_′, where F_s_ is steady-state fluorescence and F_m_′ is maximum fluorescence in the light (Maxwell and Johnson, 2000).

Total chlorophyll content was estimated non-destructively using a CI-710s SpectraVue Leaf Spectrometer (CID Bio-Science, USA) using the instrument’s reflectance-based estimation procedure (Krasavtseva and Makarov, 2024). These measurements were used to quantify drought-associated changes in photochemical performance and pigment status across treatments.

### 2.6 Tissue sampling and biochemical analyses

At harvest, shoot tissues were rinsed with distilled water, blotted dry, immediately frozen in liquid nitrogen, and stored at −80 °C until analysis. Approximately 0.1 g of fresh tissue was used for each biochemical assay.

#### 2.6.1 Hydrogen peroxide determination

Hydrogen peroxide content was quantified following the method of Velikova et al. (2000). Tissue was homogenized in 0.1% (w/v) trichloroacetic acid and centrifuged. The supernatant was reacted with potassium iodide in phosphate buffer, and absorbance was measured at 390 nm. H_2_O_2_ concentration was calculated from a standard curve and expressed as µmol g^-^¹ fresh weight.

#### 2.6.2 Determination of lipid peroxidation

Lipid peroxidation was estimated by measuring malondialdehyde (MDA) content using a thiobarbituric acid (TBA) assay adapted for a microplate reader (Kaya et al., 2022). Shoot tissue (0.1 g) was homogenized in 1 mL of 0.1% (w/v) trichloroacetic acid (TCA) on ice and centrifuged at 5500 rpm for 20 min at 4 °C. An aliquot of the supernatant was mixed with 1 mL of 0.5% (w/v) TBA prepared in 20% (w/v) TCA and incubated at 80 °C for 30 min. The reaction was immediately cooled on ice and centrifuged at 13,500 rpm for 5 min at 4 °C. Absorbance of the clarified supernatant was measured at 532 and 600 nm using a 96-well microplate reader.

#### 2.6.3 Measurement of proline content

Free proline content was quantified using the acidic ninhydrin colorimetric assay (Bates et al., 1973). Approximately 0.1 g of tissue was extracted in 3% (w/v) sulfosalicylic acid on ice, then clarified by centrifugation. An aliquot of the supernatant was reacted with glacial acetic acid and freshly prepared acidic ninhydrin reagent and incubated at 96 °C for 60 min. Reactions were terminated on ice, and proline chromophore was extracted with toluene (1 mL). The toluene phase was transferred to a fresh tube, and absorbance was measured at 520 nm using a microplate reader (200 µL; toluene blank).

### 2.7 Antioxidant enzyme extraction and assays

Enzymes were extracted from leaf tissue using 1.0 mL of ice-cold 50 mM potassium phosphate buffer (pH 7.0) supplemented with 1 mM EDTA, 1 mM D-isoascorbic acid, 2% (w/v) polyvinylpyrrolidone (PVP), and 0.05% (v/v) Triton X-100 (El-Dakak et al., 2021). Homogenates were centrifuged at 10,000 × g for 10 min at 4 °C, and the supernatants were collected as crude enzyme extracts and kept on ice for immediate analysis or stored at −20 °C until use.

#### 2.7.1 Ascorbate peroxidase

APX was determined in a UV-transparent 96-well microplate by monitoring the decrease in absorbance at 290 nm (Feng et al., 2025). The reaction mixture (200 µL final volume) contained 50 mM potassium phosphate buffer (pH 7.0), 0.2 mM EDTA, 0.5 mM D-isoascorbic acid (ascorbate), and 0.25 mM H_2_O_2_; the reaction was initiated by adding 10 µL of enzyme extract. Absorbance at 290 nm was recorded for 1 min. Blanks contained assay buffer without enzyme extract.

#### 2.7.2 Guaiacol peroxidase

Guaiacol peroxidase (GPX), used here as a proxy for class III peroxidase activity, was determined in a 96-well microplate by monitoring the increase in absorbance at 470 nm associated with guaiacol oxidation in the presence of hydrogen peroxide (Zahir et al., 2021). The reaction mixture (200 µL final volume) contained 50 mM potassium phosphate buffer (pH 7.0), 2 mM H_2_O_2_, and 2.7 mM guaiacol, and the reaction was initiated by adding 10 µL of enzyme extract. Absorbance at 470 nm was recorded for 3 min. Enzyme activities were normalized to total soluble protein concentration, which was quantified using the Bradford method with bovine serum albumin as the standard (Bradford, 1976), measuring absorbance at 595 nm. Activities are reported as U mg^-^¹ protein.

### 2.8 Statistical analysis

The pot was treated as the experimental unit. All data were analyzed using linear models with water regime, treatment group, and their interaction as fixed effects. Type II analysis of variance (ANOVA) was used for inferential screening, and model-derived means ± 95% confidence intervals are reported throughout. When significant main or interaction effects were detected, pairwise comparisons among treatment means were conducted using Tukey’s honestly significant difference (HSD) test to control for multiple comparisons (Midway et al., 2020). Correlation analyses under drought stress were performed using Spearman’s rank correlation. PCA was performed using standardized variables from treatment groups with complete biochemical datasets under drought conditions (**Supplementary Figure 1**). Linear models and ANOVA for biochemical traits were performed using the same subset of treatment groups. All statistical analyses were conducted in R (version 4.5.0).

## 3 Results

### 3.1 Senescence responses under regular watering and drought stress

**Figure 1:**
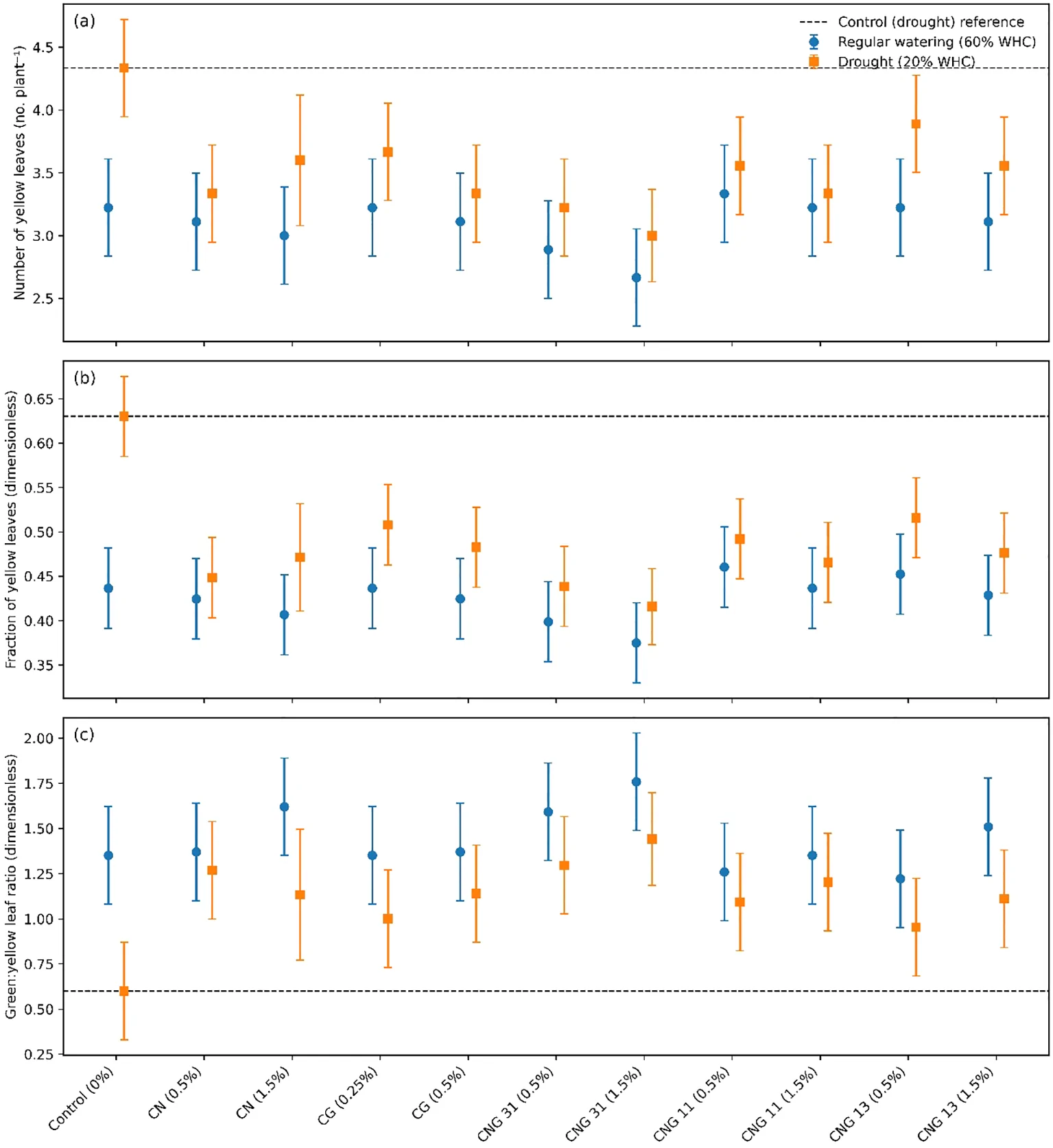
Senescence phenotype under regular watering and drought stress, model-derived means ± 95% confidence intervals for **(a)** number of yellow leaves, **(b)** fraction of yellow leaves, and **(c)** green:yellow leaf ratio. Estimates are obtained from linear models including Water × Treatment group.

Drought markedly accelerated senescence across treatments, increasing visible yellowing and shifting the green–senescent balance (**Figure 1**). Under drought, the untreated control exhibited the strongest senescence signal, with 4.33 yellow leaves pot^-^¹, a fraction yellow value of 0.63, and a green:yellow ratio of 0.60, reflecting high senescence severity across absolute senescence load (**Figure 1a**), normalized senescence intensity (**Figure 1b**), and senescence balance (**Figure 1c**). Amendment effects were comparatively small under regular watering but were clearly expressed under drought, indicating drought-contingent treatment performance.

Among formulations, CNG 31 (1.5%) most consistently reduced senescence under drought across all three indices. Relative to the droughted control, this treatment reduced the number of yellow leaves by 31% (**Figure 1a**) and lowered the fraction of yellow leaves from 0.63 to 0.416 (34% reduction; **Figure 1b**). CNG 31 (0.5%) provided a similar, slightly smaller mitigation, with a 30% reduction in fraction of yellow leaves and a corresponding increase in the green:yellow ratio. CN treatments also moderated senescence under drought, with fraction yellow reduced by 28% and green:yellow increased by 2-fold relative to the droughted control (**Figures 1b, c**), indicating consistent but less pronounced mitigation than CNG 31 (1.5%). In contrast, CG (0.5%) and several other composites showed intermediate improvements; for example, CG (0.5%) reduced the fraction of yellow leaves by 21% (**Figure 1b**), supporting formulation-dependent mitigation rather than a uniform amendment effect.

These patterns were supported statistically. Type II ANOVA indicated significant main effects of Water regime and Treatment group for all three senescence traits, and a significant Water × Group interaction specifically for the fraction of yellow leaves (**Supplementary Table 2**), confirming that treatment differences were amplified under drought and that the fraction-based metric provided the most sensitive discrimination of drought-stage senescence modulation. Among the senescence metrics, fraction of yellow leaves provided the most sensitive indication of drought-stage treatment differentiation, consistent with its normalized representation of senescence intensity across plants of different size.

### 3.2 Photosynthetic integrity and pigment responses under regular watering and drought stress

**Figure 2:**
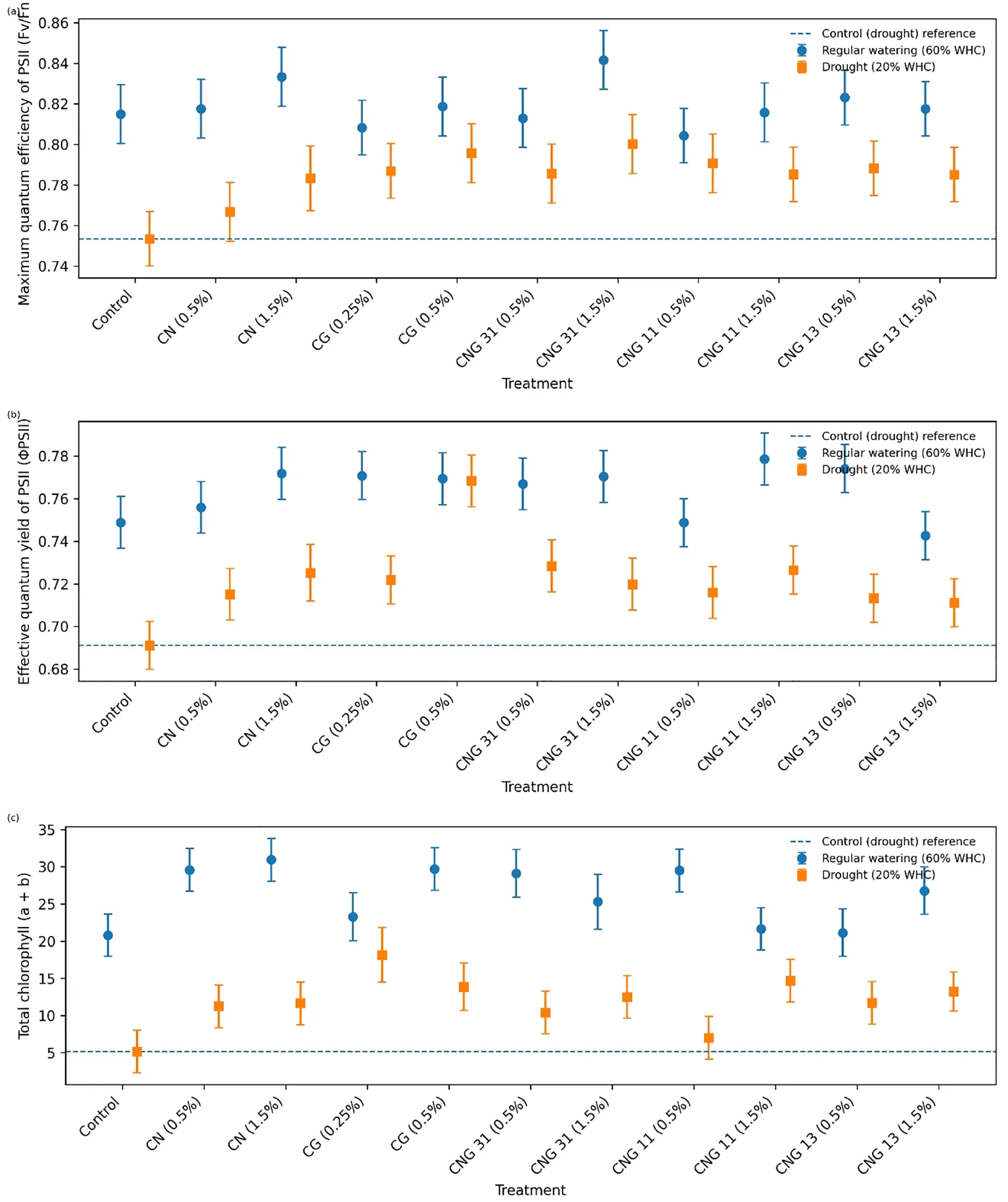
Photosynthetic integrity under regular watering and drought stress. Model-derived means ± 95% confidence intervals for **(a)** maximum quantum efficiency of PSII (F_v_/F_m_), **(b)** effective quantum yield of PSII (ΦPSII), and **(c)** total chlorophyll. Values are estimated from Water × Treatment group models.

Drought altered PSII function and pigment status across treatments, with the clearest treatment separation expressed under drought rather than under regular watering (**Figure 2**). In the untreated control, drought reduced F_v_/F_m_ from 0.815 to 0.753, reduced ΦPSII from 0.749 to 0.691, and caused a pronounced decline in total chlorophyll from 20.79 to 5.16 (75%), indicating strong pigment loss despite only moderate changes in maximum PSII efficiency (**Figures 2a–c**). Type II ANOVA detected significant Water and Group effects for all parameters, with significant Water × Group interactions for F_v_/F_m_, ΦPSII, and total chlorophyll (**Supplementary Table 2**), confirming that treatment differences were expressed most strongly under drought.

Under regular watering, F_v_/F_m_ values were high and relatively similar across treatments, consistent with intact PSII photochemistry (**Figure 2a**). Under drought, reductions in F_v_/F_m_ were smaller in most amended treatments than in the control. The highest drought-stage F_v_/F_m_ occurred in CNG 31 (1.5%), 6.2% higher than the droughted control, followed by CG (0.5%) 5.6% higher, indicating partial protection of maximum PSII efficiency under water limitation. ΦPSII showed clearer treatment separation under drought than F_v_/F_m_ (**Figure 2b**). Under drought, CG (0.5%) maintained the highest ΦPSII (11.2% above the droughted control), while CNG 31 (0.5%) (5.4% above control-S) and CN (1.5%) (5.1% above control-S) also sustained higher ΦPSII relative to the droughted control.

Total chlorophyll was the most drought-sensitive trait (**Figure 2c**). Although all treatments showed chlorophyll loss under drought, several amendments substantially attenuated depletion relative to the droughted control. CN treatments preserved chlorophyll under drought, typically maintaining levels at around 2-fold the droughted control (**Figure 2c**).

### 3.3 Oxidative stress indicators under regular watering and drought stress

#### 3.3.1 Malondialdehyde

**Figure 3:**
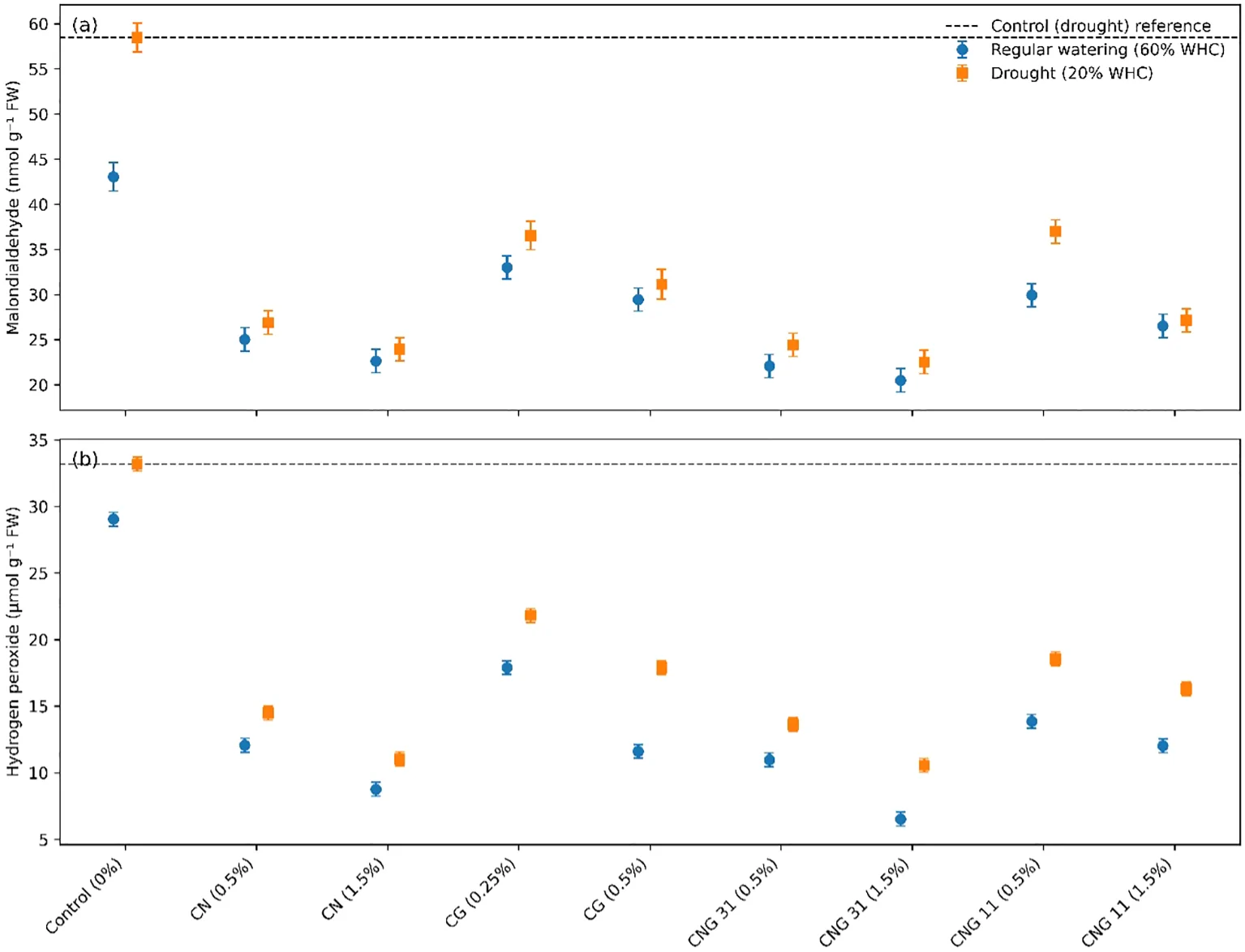
Oxidative stress indicators under regular watering and drought stress. Model-derived means ± 95% confidence intervals for **(a)** malondialdehyde and **(b)** hydrogen peroxide.

MDA content was strongly affected by both water regime and amendment treatment, with all amendments reducing MDA relative to the control under both regular watering (R) and drought stress (S) (**Figure 3a**). Under R, the control showed the highest MDA level (43.04 nmol g^-^¹ FW), whereas all amendment treatments reduced MDA. The greatest reductions were observed in CNG 31 1.5 (20.49), CNG 31 0.5 (22.08), and CN 1.5 (22.63), corresponding to decreases of approximately 52.4%, 48.7%, and 47.4%, respectively. In contrast, CG 0.25 (33.00) and several other amended treatments showed smaller reductions of approximately 23–24% relative to the control. Under S, MDA increased in most treatments, but all amended plants remained below the droughted control (58.48 nmol g^-^¹ FW). Relative to the droughted control, the strongest reductions were again observed in CNG 31 1.5 (22.52), CN 1.5 (23.95), and CNG 31 0.5 (24.41), representing decreases of approximately 61.5%, 59.0%, and 58.3%, respectively. By comparison, CNG 11 0.5, CG 0.25, and several other amended treatments showed more moderate reductions. Across water regimes, the drought-induced rise in MDA was most pronounced in the control (35.9%), followed by CNG 11 0.5 (23.6%).

#### 3.3.2 Hydrogen peroxide

H_2_O_2_ content followed a pattern similar to MDA, with the control showing the highest levels under both R and S, while all amendment treatments substantially reduced H_2_O_2_ accumulation relative to the control (**Figure 3b**). Under R, the control recorded 29.05 µmol g^-^¹ FW, and amendment treatments reduced this value. The greatest reductions were observed in CNG 31 1.5 (6.52), CN 1.5 (8.76), and CNG 31 0.5 (10.98), corresponding to decreases of about 77.6%, 69.8%, and 62.2%, respectively. Under S, H_2_O_2_ increased across all treatments, but all amendment treatments remained substantially below the droughted control (33.17 µmol g^-^¹ FW). The lowest values were again observed in CNG 31 1.5, CN 1.5, and CNG 31 0.5, reflecting reductions of about 68.2%, 66.7%, and 58.9%, respectively. In contrast, CG 0.25 and CNG 11 0.5 showed comparatively smaller reductions. Clear treatment differences were again observed under drought stress.

### 3.4 Antioxidant enzyme responses under regular watering and drought stress

#### 3.4.1 Ascorbate peroxidase activity

**Figure 4:**
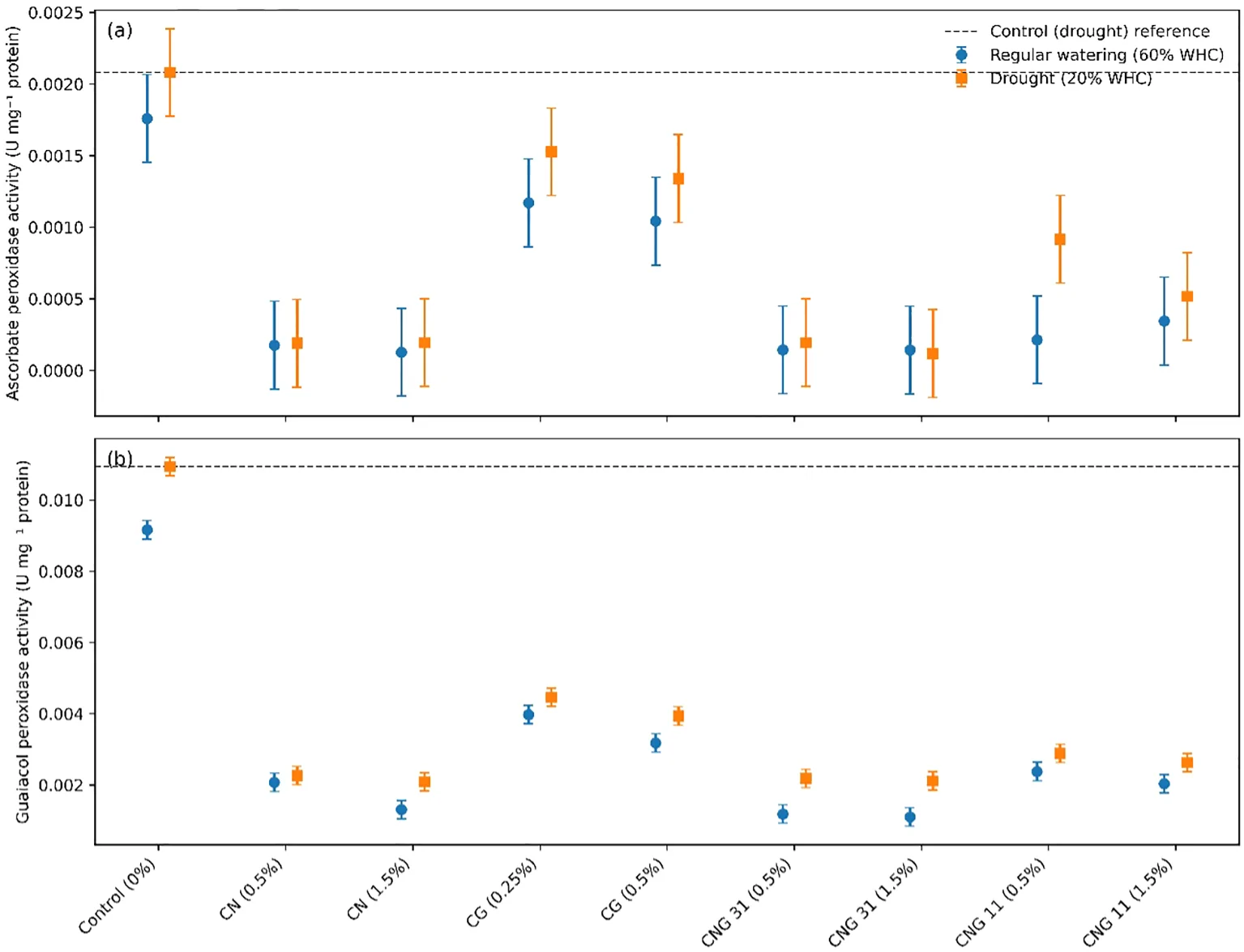
Antioxidant enzyme responses under regular watering and drought stress. Model-derived means ± 95% confidence intervals for **(a)** ascorbate peroxidase and **(b)** guaiacol peroxidase activities. Values are estimated using Water × Treatment group models.

APX activity varied markedly among treatments under both R and S conditions (**Figure 4a**). Under R, the highest APX activity was recorded in the control (0.0017 U mg^-^¹ protein), followed by CG 0.25 and CG 0.5, whereas all nanoclay-based and composite treatments showed substantially lower values, ranging from 0.0001 in CN 1.5 to low values across the composite treatments. Under regular watering, APX differed among treatments, with the control remaining higher than all nanoclay and composite formulations. Under S, APX activity remained highest in the control and in the CG treatments. In contrast, lower APX values were observed in CN 0.5, CN 1.5, CNG 31 0.5, and CNG 31 1.5. Clear treatment differences were also observed under drought stress, and the control remained higher than most nanoclay and composite treatments.

#### 3.4.2 Guaiacol peroxidase activity

GPX activity showed a different overall pattern from APX, with the control consistently exhibiting the highest values under both water regimes (**Figure 4b**). Under R, GPX in the control reached 0.0091 U mg^-^¹ protein, which was substantially higher than all amendment treatments. Among the amendments, CG 0.25 and CG 0.5 had the highest GPX values, while the lowest activities were observed in CNG 31 1.5 and CNG 31 0.5. Clear differences among treatments were observed under regular watering. Under S, GPX again reached its maximum in the control, followed by CG. Relative to R, GPX generally increased under S. The increase was 19.4% in the control and 12.3% and 23.7% in CG 0.25 and CG 0.5, respectively. Larger proportional increases were observed in CN 1.5 (59.3%), CNG 31 0.5 (83.6%), and CNG 31 1.5 (91.5%).

### 3.5 Proline accumulation under regular watering and drought stress

Proline accumulation responded strongly to water regime, with generally higher values under drought stress than under regular watering (**Supplementary Table 3**). Under regular watering, proline ranged from 692.2 µmol g^-^¹ FW in CNG 31 1.5 to 883.9 in CN 1.5, while the control recorded 697.1. Although treatment differences were evident under regular watering, pairwise differences among treatments were limited. Under drought stress, proline increased in most treatments, with the highest value recorded in CN 1.5 (1303.7 µmol g^-^¹ FW), exceeding the control (1023.7) and most other amended treatments. The drought-induced increase in proline was greatest in CN 1.5 (47.5%) and the control (46.8%), whereas smaller increases were observed in CG 0.5 (17.4%), CNG 31 0.5 (18.3%), CNG 31 1.5 (15.2%), and CNG 11 1.5 (4.4%). Notably, CNG 11 0.5 showed a slight decrease under drought (−2.7%).

### 3.6 Multivariate integration of senescence, oxidative, and antioxidant responses under drought

Biochemical traits were evaluated for the subset of treatments with complete sample availability, and the corresponding analyses reflect that subset only. To integrate coordinated drought responses across senescence, oxidative, and antioxidant traits, principal component analysis (PCA) was performed on standardized variables measured under drought conditions (**Supplementary Figure 1**). Only treatments with complete multivariate data were included in the PCA. The first two principal components explained 41.3% (PC1) and 35.3% (PC2) of the total variance, respectively. The PCA revealed a clear multivariate structure among droughted samples, reflecting combined variation in oxidative stress markers, antioxidant enzyme activities, and senescence-related traits. Treatments occupied distinct positions along the principal component axes, indicating differences in overall drought response profiles.

## 4 Discussion

Drought stress induced a coordinated shift toward oxidative imbalance, impaired photosynthetic integrity, and accelerated leaf senescence in wheat. Soil amendments based on nanoclay, calcium alginate, and their composite formulations moderated these responses under water deficit, reducing oxidative damage and delaying senescence while preserving photosynthetic function. These effects reflect attenuation of drought-induced stress cascades rather than complete stress avoidance, indicating that amendment treatments buffer oxidative and senescence processes downstream of water limitation (Nyaupane et al., 2024).

### 4.1 Drought-induced senescence as an integrative stress phenotype

Leaf senescence is a regulated developmental program that is frequently accelerated by drought via redox- and hormone-linked signalling, where enhanced ROS production promotes chlorophyll degradation and chloroplast dismantling, thereby shortening the duration of effective canopy photosynthesis (Guo et al., 2021). In this study, senescence indices showed strong drought sensitivity and clear treatment dependence, with the fraction of yellow leaves providing the most discriminating endpoint (significant Water × Group interaction). This metric is particularly robust because it normalizes senescence intensity to total leaf number, reducing confounding from treatment effects on plant size or leaf production and indicating that amendment effects were expressed primarily by altering the drought-stage senescence trajectory rather than baseline leaf aging.

Among formulations, CNG 31 (1.5%) consistently moderated drought-stage senescence across absolute (yellow leaf number) and normalized (fraction yellow, green: yellow ratio) indices, supporting delayed canopy deterioration under water deficit. These treatment-related differences in plant appearance and senescence under drought were also evident from representative photographs of wheat plants (**Supplementary Figure 2**), illustrating the greater retention of green leaf tissue in selected amendment treatments compared with the droughted control. Such moderation is consistent with prolonged retention of functional green tissue, a phenotype frequently associated with sustained canopy function under stress and often discussed within a stay-green framework in wheat (Moraga et al., 2022; Abbas et al., 2025). The weaker and more variable responses observed for CN-only treatments and CG/composite variants further indicate that senescence buffering is formulation- and dose-dependent, reinforcing the need for optimization rather than assuming uniform benefit across amendment types and rates (Georgieva and Vassileva, 2023).

### 4.2 Preservation of photosynthetic integrity under drought

The fluorescence and pigment responses indicate that drought reduced photosynthetic integrity primarily through pigment loss and operational downregulation, while differences in chronic PSII damage were comparatively limited. This is consistent with established fluorescence interpretation: F_v_/F_m_ is a conservative indicator of maximal PSII efficiency and typically shows large declines mainly under severe or prolonged stress that induces sustained photoinhibition, whereas ΦPSII is more responsive to dynamic regulation of photochemistry under illumination and therefore often separates treatments more clearly under moderate drought (de Sampaio-Filho et al., 2026).

Control exhibited a modest drop in F_v_/F_m_ (8%) but a very large chlorophyll loss (75%), indicating that drought strongly affected pigment status and canopy greenness even though maximal PSII efficiency was not catastrophically impaired. Treatments that maintained higher F_v_/F_m_ under drought, most notably CNG 31 (1.5%) and CG (0.5%) therefore likely reduced the extent of sustained PSII down-regulation or damage, consistent with partial stress mitigation rather than complete stress avoidance. ΦPSII provided clearer functional discrimination under drought, with CG (0.5%) showing the strongest preservation of operating photochemical efficiency. Because ΦPSII reflects the balance between photochemical energy use and dissipation under light, its higher drought-stage values in selected treatments are consistent with improved maintenance of electron transport under stress and/or reduced need for protective downregulation. Similar protective effects have been reported when antioxidant capacity and osmotic balance are maintained (Abdo et al., 2024; Todorova et al., 2024; Pecka et al., 2025). Preservation of photosynthetic efficiency is a hallmark of drought tolerance, linked to reduced ROS accumulation and improved electron transport under stress (Qiao et al., 2024).

Total chlorophyll showed the largest drought sensitivity and the strongest treatment divergence. The pronounced chlorophyll depletion in the droughted control is consistent with accelerated senescence and chloroplast dismantling under water deficit, processes that are closely linked to oxidative stress and redox imbalance during drought. The strong pigment retention in CG (0.25%) and CNG 11 (1.5%) suggests that certain amendment configurations delayed pigment breakdown and maintained leaf “structural capacity” for photosynthesis. However, pigment retention did not perfectly rank with ΦPSII, which is physiologically expected: maintaining chlorophyll preserves light-harvesting capacity, but realized photochemical performance under drought is additionally constrained by stomatal, mesophyll, and biochemical limitations (Li et al., 2025).

The dataset supports a realistic stress-mitigation framework: amendments moderated drought impacts on PSII function and pigment stability, with ΦPSII and chlorophyll being more sensitive discriminators of treatment performance than F_v_/F_m_, consistent with moderate drought where limitations to photosynthesis are frequently dominated by diffusive/metabolic constraints rather than widespread chronic PSII photodamage. Several amendment treatments, mainly CNG 31, required lower water addition than the untreated control during drought. Under drought, the untreated control required 106.0 g pot^-^¹ across recorded weighing dates, whereas CNG 31 (0.5%) and CNG 31 (1.5%) required 81.1 and 61.7 g pot^-^¹, respectively, corresponding to 23.5% and 41.8% lower recorded water addition. These values represent water added to restore pots to target WHC and should not be interpreted as direct measurements of evaporation, transpiration, soil hydraulic conductivity, or field-scale water productivity. Nevertheless, they provide useful pot-scale context consistent with the possibility that amendment-induced changes in the soil–plant water environment contributed to the improved physiological performance observed under drought.

### 4.3 Oxidative stress mitigation and antioxidant regulation

MDA is widely used as an indicator of membrane lipid peroxidation in plants, and higher MDA generally reflects greater oxidative damage to cellular membranes (Mohagheghian et al., 2025). In the present study, the consistently highest MDA values in the control under both R and S, together with the strong drought-induced increase under S, indicate that untreated plants experienced the greatest membrane-level oxidative injury. This pattern is fully consistent with the established view that drought enhances ROS generation, promotes lipid peroxidation, and raises MDA accumulation in wheat and other crop plants (Aliyeva et al., 2023; Ishfaq et al., 2024; Keke et al., 2025).

All amendment treatments reduced MDA relative to the control under both water regimes, but the magnitude of reduction differed considerably among formulations. Under drought stress, the lowest MDA values were recorded in CNG 31 1.5, CN 1.5, and CNG 31 0.5, indicating better control of lipid peroxidation under stress, whereas the stronger increase in CNG 11 0.5 suggests greater membrane sensitivity in that formulation. Similar treatment-dependent changes in MDA have been reported in wheat under drought, where lower MDA is generally associated with improved oxidative protection and stress tolerance (Mokhtari et al., 2024). More broadly, the separation between the better-performing nanoclay-based treatments and the less responsive formulations indicates that oxidative outcome depended not only on amendment presence but also on the specific nanoclay–alginate ratio and dose. This interpretation is in line with broader evidence that nanomaterial-based drought mitigation can reduce MDA and oxidative injury, but that the magnitude and direction of the effect depend strongly on material type, dose, and formulation (Ahluwalia et al., 2021; Chandrashekar et al., 2023).

H_2_O_2_ occupies a dual position in plant stress physiology: it is both a damaging ROS when excessively accumulated and a relatively stable signaling molecule involved in stress acclimation (Sachdev et al., 2021; Fedoreyeva, 2024). In this study, the high H_2_O_2_ levels in the control under both R and S, and the further increase under drought, indicate a substantially greater oxidative stress in untreated plants. All amendments lowered H_2_O_2_ levels relative to the control, both under R and S conditions, indicating that amendment application consistently reduced cellular peroxide accumulation. The lowest H_2_O_2_ concentrations were found in CNG 31 1.5, CN 1.5, and CNG 31 0.5, whereas CG 0.25 and CG 0.5 remained relatively higher. In wheat, reduced H_2_O_2_ accumulation under stress has previously been linked with improved drought acclimation and lower membrane damage, whereas sustained high peroxide levels are usually associated with a poorer oxidative response (Abid et al., 2018; Ahmad et al., 2018; Habib et al., 2020).

### 4.4 APX responses under R and S

APX activity was strongly influenced by both water regime and amendment type. The relatively high APX activity in the control under both R and S is consistent with the established role of APX as a key H_2_O_2_-detoxifying enzyme in the ascorbate–glutathione cycle under drought-induced oxidative stress (Mishra et al., 2023). In wheat, APX activity commonly changes under water deficit, although the magnitude and direction of the response can differ depending on genotype, stress severity, and recovery status. Studies in wheat have shown that drought can enhance APX activity as part of antioxidant defense, while isoform-specific and cultivar-specific differences are also common (Tyagi et al., 2020; Sohail et al., 2026).

The comparatively high APX activity in CG 0.25 and CG 0.5, although still lower than the control, suggests that calcium alginate-treated plants maintained a relatively strong APX-based antioxidant response under both water regimes. In contrast, the markedly lower APX values in CN and most composite formulations indicate that these amendments altered antioxidant regulation differently from calcium alginate alone. Such differences are plausible because antioxidant responses under drought are highly dynamic and depend on how strongly a treatment modifies cellular redox balance, water status, and downstream ROS-scavenging requirements. Similar variability in APX behavior under water deficit has also been reported in recent wheat studies, where drought increased APX but the degree of induction varied among genetic backgrounds and treatment conditions (Mokhtari et al., 2024; Lamlom et al., 2025).

### 4.5 GPX responses under R and S

Unlike APX, GPX showed a more uniform pattern, with the control remaining higher than all amended treatments under both R and S. In plant physiological studies, guaiacol peroxidase is generally used as a proxy for class III peroxidase activity, a multifunctional enzyme system involved in H_2_O_2_ metabolism, cell wall-related oxidative processes, and stress responses (Li et al., 2024b). Class III peroxidases are known to respond to abiotic stress in a pathway-specific manner and can show substantial functional diversification (Aleem et al., 2022; Li et al., 2024b). Within the amended treatments, CG retained the highest GPX values, whereas CNG 31 remained among the lowest under both water regimes. However, the strong drought-induced increase in GPX in CNG 31 0.5 and CNG 31 1.5, despite their low absolute values under R, contrasted with the APX response of the same treatments, particularly CNG 31 1.5, where APX declined while GPX increased sharply. A similar divergence was observed in selected amended treatments, where GPX decreased slightly under S while APX increased. These differences suggest that APX and GPX were not co-regulated in the same way across treatments. This is consistent with previous work showing that drought responses of antioxidant enzymes in wheat can be pathway-specific, with distinct enzymes or isoforms responding differently depending on tissue status and oxidative conditions (Mokhtari et al., 2024).

Overall, the GPX data indicate that drought-induced antioxidant responses depended not only on whether an amendment was applied, but also on the specific enzymatic pathway involved. APX and GPX were therefore interpreted as selected indicators of H_2_O_2_ detoxification and peroxidase-related redox metabolism, rather than as a complete representation of the antioxidant system. Because other antioxidant components, including superoxide dismutase, catalase, glutathione reductase, ascorbate–glutathione metabolites, and non-enzymatic antioxidants, were not measured, conclusions regarding antioxidant regulation were restricted to the APX and GPX pathways assessed in this study. Future work should incorporate a broader antioxidant profile to determine whether nanoclay–alginate amendments modulate redox homeostasis through coordinated changes across multiple enzymatic and non-enzymatic defense systems (Tyagi et al., 2020).

### 4.6 Proline accumulation and osmotic regulation

Proline accumulation increased under drought stress in most treatments, consistent with the well-documented role of proline in plant drought responses. In wheat and other cereals, proline commonly accumulates under water deficit and contributes to osmotic regulation, maintenance of cell turgor, and stabilization of stress-affected metabolism, although its magnitude varies with genotype, stress duration, and the broader physiological condition of the plant (Wahab et al., 2022; Li et al., 2024a). The strong increase in proline in the control under drought stress, together with the similarly large increase in CN 1.5, indicates a pronounced osmotic response in these treatments. CN 1.5 showed the highest absolute proline level under drought, suggesting that the higher nanoclay dose was associated with a strong osmotic response rather than suppression of compatible solute accumulation. By contrast, several composite treatments, especially CNG 31 1.5 and CNG 11 1.5, showed comparatively small increases, while CNG 11 0.5 showed a slight decrease under drought. This relatively stable proline response in CNG 11 suggests that this formulation may have relied more strongly on enzymatic antioxidant responses than on additional proline accumulation. More broadly, the contrast between CN 1.5 and several composite formulations indicates that nanoclay–alginate combinations did not behave uniformly, and that their effects depended on formulation ratio and dose. This is consistent with the wider drought literature, which shows that osmotic response is an important but variable component of wheat stress adaptation rather than a universally proportional response (Sun et al., 2025).

Collectively, the results indicate that amendment-mediated drought mitigation was not associated with a single physiological pathway but rather with coordinated regulation of interconnected stress responses. Treatments that maintained lower H_2_O_2_ and MDA concentrations under drought generally exhibited greater chlorophyll retention, improved PSII performance, and reduced senescence development. This convergence of responses suggests that the moderation of oxidative stress helped preserve chloroplast function and delay drought-induced deterioration of leaf tissues. The stronger responses observed in selected formulations, particularly CNG 31, further indicate that amendment composition influences the extent to which these protective effects are expressed under water limitation.

### 4.7 Implications and future directions

The present study demonstrates that drought mitigation by nanoclay-, alginate-, and composite-based amendments involves coordinated regulation of multiple stress-related processes rather than isolated effects on individual physiological or biochemical traits. Across treatments, reduced oxidative damage was associated with improved photosynthetic performance, greater chlorophyll retention, and delayed senescence, indicating that preservation of functional leaf activity was a key component of the observed drought resilience. The stronger and more consistent responses of selected composite formulations further highlight the importance of amendment composition and dosage in determining treatment effectiveness under water limitation.

These findings highlight the potential of nanoclay–alginate formulations as multifunctional drought-mitigation amendments. However, the present results were obtained under controlled greenhouse conditions and should therefore be validated under field conditions and across a broader range of soils, wheat genotypes, and drought scenarios. Field performance may differ because of environmental variability, soil heterogeneity, irrigation method, rooting depth, microbial activity, and amendment persistence under repeated drying–rewetting cycles. Root traits were not measured in the present study; therefore, the contribution of root architecture, root biomass allocation, soil–root contact, and rhizosphere hydraulic processes to the observed drought-mitigation responses could not be directly determined. In addition, direct plant water-status indicators, such as relative water content, leaf water potential, or osmotic potential, were not assessed; therefore, drought responses were interpreted based on the imposed WHC levels together with senescence, photosynthetic, oxidative, antioxidant, and proline responses. Future studies integrating physiological and biochemical assessments with measurements of soil hydraulic characteristics, plant water status, root responses, amendment longevity, and yield-related traits will provide a more comprehensive understanding of the mechanisms governing amendment performance and support optimization of formulation design for agricultural applications under water-limited conditions.

## 5 Conclusions

Drought stress induced a coordinated deterioration of wheat performance, expressed as accelerated leaf senescence, chlorophyll depletion, reduced PSII efficiency, increased H_2_O_2_ and MDA accumulation, and shifts in antioxidant enzyme activity and proline content. Among the evaluated formulations, CNG 31, particularly at 1.5%, most consistently moderated drought-induced senescence and oxidative damage, while CG treatments were more effective in preserving chlorophyll and photochemical performance. CN 1.5 and CNG 31 also showed the lowest drought-stage MDA and H_2_O_2_ levels, indicating stronger control of oxidative injury relative to the droughted control.

The antioxidant response was strongly treatment dependent. APX and GPX did not show a uniform drought pattern across formulations, indicating that amendment-mediated drought mitigation was associated with differential regulation of redox metabolism rather than simple upregulation of antioxidant activity. In contrast, proline accumulation was driven mainly by water regime, with weaker treatment discrimination, suggesting that proline reflected osmotic adjustment under drought but was less informative than oxidative and senescence traits for distinguishing amendment performance.

Taken together, the results show that drought mitigation in this system depended on the coordinated regulation of senescence, photosynthetic function, oxidative balance, and metabolic adjustment. These findings emphasize that evaluating drought tolerance requires simultaneous consideration of senescence, photosynthesis, redox homeostasis, and metabolic regulation. Overall, the study provides a framework for linking physiological and biochemical traits to drought response in wheat and offers a basis for future evaluation and optimization of nanoclay–alginate soil amendments for water-limited cropping systems.

## Data Availability

The original contributions presented in the study are included in the article/**Supplementary Material**. Further inquiries can be directed to the corresponding author.
