# Supplementary material for: Nanoclay- and alginate-based soil amendments preserve photosynthetic function, delay senescence, and reduce oxidative damage in drought-stressed wheat
Source: Front Plant Sci. 2026 Jul 27;17:1842102. doi: 10.3389/fpls.2026.1842102 (PMC13453755; doi:10.3389/fpls.2026.1842102)
Supplement: Supplementary file 1 [file Supplementaryfile1.docx]

**Supplementary document**

**Table S1.** Properties of CN, CG, and composite formulations.

| **Property** | **CN** | **CG** | **CNG 11** | **CNG 13** | **CNG 31** |
| --- | --- | --- | --- | --- | --- |
| BET surface area (m² g⁻¹) | 109.2 | 41.4 | 118.8 | 102.3 | 139.6 |
| Total pore volume (cm³ g⁻¹) | 0.0699 | 0.0301 | 0.0853 | 0.0603 | 0.0933 |
| Mean pore radius (nm) | 1.31 | 1.55 | 1.51 | 1.17 | 1.44 |
| Particle size (nm) | 500 | 841 | 584 | 714 | 548 |

**Table S2.** Type II ANOVA summary for senescence, photosynthetic, biochemical, and antioxidant traits under regular watering and drought stress.

| **Trait** | **Effect** | **df** | **F** | **p** |
| --- | --- | --- | --- | --- |
| Number of yellow leaves | Water | 1 | 25.083 | <0.001 |
|  | Group | 10 | 3.330 | <0.001 |
|  | Water × Group | 10 | 1.028 | 0.361 |
| Fraction of yellow leaves | Water | 1 | 37.274 | <0.001 |
|  | Group | 10 | 5.139 | <0.001 |
|  | Water × Group | 10 | 2.109 | 0.004 |
| Green:yellow leaf ratio | Water | 1 | 28.657 | <0.001 |
|  | Group | 10 | 3.215 | <0.001 |
|  | Water × Group | 10 | 0.869 | 0.544 |
| Maximum quantum efficiency of PSII (Fv/Fm) | Water | 1 | 131.380 | <0.001 |
|  | Group | 10 | 3.629 | <0.001 |
|  | Water × Group | 10 | 2.051 | 0.010 |
| Effective quantum yield of PSII (ΦPSII) | Water | 1 | 275.116 | <0.001 |
|  | Group | 10 | 10.141 | <0.001 |
|  | Water × Group | 10 | 3.779 | <0.001 |
| Total chlorophyll | Water | 1 | 118.904 | <0.001 |
|  | Group | 10 | 4.802 | <0.001 |
|  | Water × Group | 10 | 2.941 | <0.001 |
| Malondialdehyde (MDA) | Water | 1 | 198.773 | <0.001 |
|  | Group | 8 | 9.214 | <0.001 |
|  | Water × Group | 8 | 4.667 | <0.001 |
| Hydrogen peroxide (H₂O₂) | Water | 1 | 176.542 | <0.001 |
|  | Group | 8 | 8.541 | <0.001 |
|  | Water × Group | 8 | 3.923 | 0.002 |
| Ascorbate peroxidase activity (APX) | Water | 1 | 28.443 | <0.001 |
|  | Group | 8 | 5.712 | <0.001 |
|  | Water × Group | 8 | 2.842 | 0.011 |
| Guaiacol peroxidase activity (GPX) | Water | 1 | 34.921 | <0.001 |
|  | Group | 8 | 6.081 | <0.001 |
|  | Water × Group | 8 | 3.012 | 0.009 |
| Proline | Water | 1 | 62.504 | <0.001 |
|  | Group | 8 | 4.926 | <0.001 |
|  | Water × Group | 8 | 1.847 | 0.086 |

Type II ANOVA was performed using linear models with water regime, treatment group, and their interaction (Water × Group) as fixed effects. Biochemical traits were assessed for a subset of treatments due to sample availability; analyses reflect available data only. df, F, and p-values are reported for screening and inference.

**Table S3.** Proline concentration under regular watering and drought stress.

| **Treatment** | **Rate (%)** | **Regular watering (µmol g⁻¹ FW)** | **Drought stress (µmol g⁻¹ FW)** |
| --- | --- | --- | --- |
| Control | 0.0 | 697.13 ± 41.27 | 1023.72 ± 144.71 |
| CN | 0.5 | 841.62 ± 57.61 | 1030.52 ± 59.34 |
| CN | 1.5 | 883.86 ± 52.65 | 1303.71 ± 35.83 |
| CG | 0.25 | 804.22 ± 52.97 | 991.36 ± 51.33 |
| CG | 0.5 | 777.72 ± 45.59 | 912.69 ± 45.69 |
| CNG 31 | 0.5 | 738.46 ± 38.11 | 873.23 ± 31.28 |
| CNG 31 | 1.5 | 692.23 ± 32.66 | 795.75 ± 17.18 |
| CNG 11 | 0.5 | 881.52 ± 57.49 | 857.83 ± 18.45 |
| CNG 11 | 1.5 | 758.85 ± 19.45 | 792.61 ± 26.92 |

Values are means ± standard error (SE) of biological replicates.

**Figure S1**. Principal component analysis of biochemical and senescence traits under drought stress. PCA was performed on standardized biochemical and senescence variables measured under drought conditions. Each point represents an individual sample, with treatment groups indicated by color. The first two principal components explain 41.3% (PC1) and 35.3% (PC2) of the total variance. Only treatments with complete multivariate data under drought were included.

**Figure S2.** Representative images of wheat plants grown under drought stress showing the effects of calcium alginate (CG), nanoclay (CN), and nanoclay–alginate composite formulations (CNG 11, CNG 13, and CNG 31) compared with the untreated control
